# Anti-Inflammatory Potential of* Carpomitra costata* Ethanolic Extracts via Inhibition of NF-*κ*B and AP-1 Activation in LPS-Stimulated RAW264.7 Macrophages

**DOI:** 10.1155/2018/6914514

**Published:** 2018-02-26

**Authors:** Mi-Jin Yim, Jeong Min Lee, Grace Choi, Dae-Sung Lee, Won Sun Park, Won-Kyo Jung, Saegwang Park, Su-Kil Seo, Jungik Park, Il-Whan Choi, Sun Young Ma

**Affiliations:** ^1^National Marine Biodiversity Institute of Korea, Seocheon 33662, Republic of Korea; ^2^Department of Physiology, Kangwon National University School of Medicine, Chuncheon 24341, Republic of Korea; ^3^Department of Biomedical Engineering and Center for Marine-Integrated Biomedical Technology (BK21 Plus), Pukyong National University, Busan 48513, Republic of Korea; ^4^Department of Microbiology and Immunology, Inje University College of Medicine, Busan 49267, Republic of Korea; ^5^Division of Endocrinology and Metabolism, Department of Internal Medicine, Dong-Eui Medical Center, Busan 47223, Republic of Korea; ^6^Department of Radiation Oncology, College of Medicine, Kosin University, Busan 47392, Republic of Korea

## Abstract

Marine algae have valuable health and dietary benefits. The present study aimed to investigate whether an ethanol extract of* Carpomitra costata* (CCE) could inhibit the inflammatory response to LPS. CCE attenuated the production of proinflammatory mediators, such as prostaglandin E_2_ (PGE_2_) and nitric oxide (NO), by inhibiting inducible nitric oxide synthase (iNOS) and cyclooxygenase-2 (COX-2) expression in LPS-induced RAW264.7 macrophages. CCE also inhibited the expression of proinflammatory cytokines such as IL-1*β*, TNF-*α*, and IL-6. CCE suppressed the LPS-induced DNA-binding activity of (NF-*κ*B) and activator protein-1 (AP-1). In addition, CCE attenuated the LPS-stimulated phosphorylation of c-Jun N-terminal kinase/stress-activated protein kinase (JNK) and phosphatidylinositol 3′-kinase/Akt (PI3K/Akt). Functional aspects of the JNK and Akt signaling pathways were analyzed using specific inhibitors, which attenuated the LPS-induced production of proinflammatory cytokines, and NO and PGE_2_ expression by suppressing AP-1 and NF-*κ*B activity. In particular, the AP-1 signaling pathway is not involved in the production of inflammatory cytokines, such as IL-6, TNF-*α*, and IL-1*β*. These results suggested that CCE might exert its anti-inflammatory action by downregulating transcriptional factors (NF-*κ*B and AP-1) through JNK and Akt signaling pathways. The current study suggested that CCE might be a valuable candidate for the treatment of inflammatory disorders.

## 1. Introduction

Marine algae have long been used as potential medicinal and dietary sources in Asia. Algae are rich in vitamins, minerals, dietary fibers, essential fatty acids, enzymes, polysaccharides, and various functional polyphenols [1]. The brown seaweed* Carpomitra costata* (Stackhouse) Batters (Sporochnaceae) has been part of the traditional diet in Asia. A previous study reported that* C. costata* extracts (CCE) have antibacterial, antifungal, and photoprotective effects [2, 3].

Inflammation is a defensive reaction in response to different stimuli, such as infection, chemical exposure, tissue damage, or other foreign substances [4]. However, excessive or inappropriate inflammatory responses can cause numerous chronic inflammatory disorders, including inflammatory bowel disease, cancer, atherosclerosis, and diabetes [5, 6]. Inflammation is mediated by inflammatory mediators that are generated primarily by macrophages that have been overactivated by various intrinsic or extrinsic stimuli that initiate and maintain immune responses [7]. Thus, the regulation of macrophage activation might have therapeutic potential for the treatment of many inflammatory disorders. Lipopolysaccharide (LPS), a major component of Gram-negative bacteria cell walls, induces a number of cellular responses that act as extremely strong stimulators of innate or natural immunity [8]. Therefore, LPS stimulation of macrophages is a powerful model for the study of inflammation that is mediated by inflammatory mediators from activated macrophages.

During the inflammatory process, large amounts of inflammatory mediators are produced. Among them, nitric oxide (NO) and prostaglandin E_2_ (PGE_2_) are products of the inducible isoforms of inducible nitric oxide synthase (iNOS) enzymes and cyclooxygenase- (COX-) 2, respectively [9, 10]. NO produced by iNOS has been reported to be a primary biological messenger that is involved in diverse physiological activities, including neural communication, vasodilation, and host defense, and also has beneficial antiparasitic, antiviral, microbiocidal, and antitumoral effects [9]. However, excessive and sustained NO release can damage the host and has been correlated with the progression of many inflammatory diseases. Prostaglandins (PGs) play a critical role in the generation of the inflammatory response and its expression is increased in inflamed tissue. PG, which is synthesized by COX-2, is another important mediator in the pathogenesis of inflammation. COX-2 catalyzes the conversion of arachidonic acid to prostaglandin H_2_, the precursor of a variety of biologically active mediators, such as PGE_2_ [11, 12]. Additionally, large amounts of inflammatory cytokines [interleukin-1*β* (IL-1*β*), interleukin-6 (IL-6), and tumor necrosis factor alpha (TNF-*α*)] are released during the response of tissue to injury, resulting in systemic responses to the inflammatory process.

Therefore, controlling inflammatory mediators may have therapeutic applications for the treatment of various inflammatory disorders. In the present study, to evaluate the anti-inflammatory potential of CCE, we investigated whether CCE could attenuate the production of NO, PGE_2_, and proinflammatory cytokines that are important targets in the treatment of inflammatory disorders. In addition, we determined the intracellular signaling pathways responsible for the regulatory actions of CCE in mouse RAW264.7 macrophages that are stimulated by LPS.

## 2. Materials and Methods

### 2.1. Chemicals and Materials

We purchased LPS (*Escherichia coli* O111:B4) and U0126 (cat. number U120) from Sigma Chemical Co. (St. Louis, MO, USA). SP600125 (cat. number BML-EI305) and SB203580 (cat. number BML-EI286) were purchased from Enzo Life Sciences, Inc. (Farmingdale, NY, USA). LY294002 (cat. number 440202) was purchased from Calbiochem (La Jolla, CA, USA). SR11302 (cat. number SR-11032) was purchased from R&D Systems (Minneapolis, MN, USA). BAY 11-7082 and parthenolide were purchased from Santa Cruz Biotechnology (Santa Cruz, CA, USA). Antibodies against c-Jun N-terminal kinase (JNK) (cat. number 9252), phospho- (p-) JNK (cat. number 9251), p-extracellular signal-related kinase (ERK) (cat. number 9106), Akt (cat. number 9272), p-Akt (cat. number 4058), and p-p38 mitogen-activated protein kinase (MAPK) (cat. number 9211) were purchased from Cell Signaling Technology, Inc. (Danvers, MA, USA). Antibodies against p38 MAPK (cat. number sc-535), ERK (cat. number sc-94), iNOS (cat. number sc-651), and COX-2 (cat. number sc-1745) were purchased from Santa Cruz Biotechnology.

### 2.2. Preparation of the Ethanol Extract from* C. costata*

The brown seaweed,* C. costata,* was collected near Ulleung Island, Korea. After collection,* C. costata* was washed with tap water to remove slats, epiphytes, and sand attached to the surface of the samples and then stored at −20°C. The frozen samples were lyophilized and homogenized using a grinder before extraction. The dried powder was extracted with 70% EtOH (1 : 10 w/v) for 1 h (five times) by sonication, and then the extract was evaporated in vacuo. The extract was dissolved in DMSO before use in the experiment.

### 2.3. Cell Culture

RAW264.7 macrophages obtained from the American Type Culture Collection (Manassas, VA, USA) were cultured in DMEM containing 10% fetal bovine serum, 100 U/mL of penicillin, and 100 *μ*g/mL of streptomycin at 37°C in a 5% CO_2_ humidified air environment.

### 2.4. Cell Viability Assay

Cell viability was assessed using the Cell Counting Kit-8 (CCK-8, Dojindo Laboratories, Kumamoto, Japan) method. Briefly, 96 wells containing 8 × 10^4^ cells/mL were treated with CCE (30–100 *μ*g/mL) and the cell culture plate was incubated for 24 h. CCK-8 was then added to each well and the plate was again incubated at 37°C for 1 h. The absorbance of each well was recorded at 450 nm using a microplate reader (SpectraMax M2e, Molecular Devices, Sunnyvale, CA, USA).

### 2.5. Nitric Oxide Assay

After cells (5 × 10^5^ cells/ml) were treated with 100 ng/mL LPS alone or with 30, 50, or 100 *μ*g/mL CCE in 24-well plates for 24 h, 100 *μ*L of each culture medium was mixed with the same volume of Griess reagent. Nitrite levels were determined at 540 nm using an enzyme-linked immunosorbent assay (ELISA) plate reader (SpectraMax M2e).

### 2.6. Measurement of PGE_2_

RAW264.7 cells were cultured in 6-well plates (5 × 10^5^ cells/mL) and incubated with CCE in the presence or absence of LPS (100 ng/mL) for 24 h. The culture medium was collected for determination of the PGE_2_ concentration by ELISA (Cayman Chemical, Ann Arbor, MI, USA).

### 2.7. Western Blot Analysis

The cells (3.2 × 10^5^ cells/60 mm culture dish plate) were washed three times with 50 *μ*L PBS and lysed with lysis buffer (Mammalian Cell-PE LB™, G-Biosciences, St. Louis, MO, USA). The proteins were separated on polyacrylamide mini-gels and transferred to nitrocellulose membranes (GE Healthcare Life Sciences, Chalfont, UK). Following overnight incubation with the appropriate primary antibody, the membranes were incubated with a secondary antibody conjugated to horseradish peroxidase. The immunoreactive bands were visualized using an ECL detection system (Pierce Biotechnology, Inc., Rockford, IL, USA).

### 2.8. Enzyme-Linked Immunosorbent Assay (ELISA)

The cytokine levels in the cell culture medium were assessed using ELISA. ELISA kits purchased from BioLegend (San Diego, CA, USA) were used to measure the levels of IL-6 and TNF-*α*, and a kit obtained from R&D Systems (Minneapolis, MN) was used to measure the IL-1*β* levels. The absorbance was measured at 450 nm using a microplate reader (SpectraMax M2e).

### 2.9. Electrophoretic Mobility Shift Assay (EMSA)

Nuclear extract was prepared using the NE-PER nuclear extraction reagent (Pierce Biotechnology, Inc.). An oligonucleotide containing the immunoglobulin *κ*-chain binding site (*κ*B, 5′-GATCTCAGAGGGGACTTTCCGAGAGA-3′) and activator protein-1 (AP-1) DNA-binding site (5′-CGCTTGATGACTCAGCCGGAA-3′) was synthesized as a probe for the DNA-protein binding assay. The 3′ end of the probe was labeled with biotin using the biotin 3′-end DNA labeling kit (Pierce Biotechnology, Inc.). The binding reactions contained 5 *μ*g nuclear extract protein, buffer, 50 ng poly (dI-dC), and 20 fM biotin-labeled DNA. The reaction mixture was incubated for 20 min at room temperature. The competition reactions were performed by the addition of 100-fold excess of unlabeled NF-*κ*B p65 and AP-1 to the reaction mixture. The mixture was then separated using electrophoresis on a 5% polyacrylamide gel in 0.5x Tris-borate buffer and transferred to nylon membranes. The biotin-labeled DNA was detected using a LightShift Chemiluminescent EMSA kit (Pierce Biotechnology, Inc.).

### 2.10. Statistical Analysis

Data values are presented as the mean ± SEM All statistical analyses were performed with GraphPad Prism software 5.0 (GraphPad Software Inc., La Jolla, CA, USA). Comparisons between groups were performed by Dunnett's multiple range tests. Differences were considered statistically significant at *P* < 0.05.

## 3. Results

### 3.1. Effects of CCE on the Viability of RAW264.7 Macrophages

**Figure 1:**
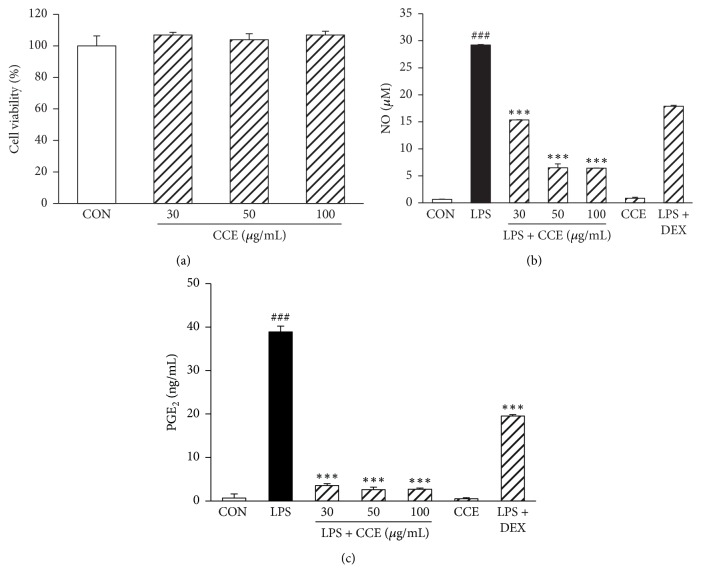
Effect of CCE on LPS-induced NO and PGE_2_ production in RAW264.7 macrophages. (a) RAW264.7 cells were pretreated with the indicated concentrations of CCE (30–100 *μ*g/mL) for 24 h. Cell viability was assessed using the CCK-8 assay and the results are expressed as the percentage of surviving cells relative to control cells (DMSO-treated). (b and c) The cells were pretreated with CCE (30–100 *μ*g/mL) for 1 h and stimulated with LPS (100 ng/mL) for 24 h. Levels of NO and PGE_2_ in the supernatant were detected using the Griess reaction assay (b) and ELISA (c). Each value indicates the mean ± SEM and is representative of the results obtained from three independent experiments. ^###^*P* < 0.001 versus control group; ^*∗∗∗*^*P* < 0.001 versus LPS-stimulated group. DEX: dexamethasone.

We first evaluated the cytotoxicity of CCE using CCK-8 assays. CCK-8 assays were performed in cells incubated with CCE for 24 h. At concentrations of ≤100 *μ*g/mL, CCE did not affect cell viability (Figure 1(a)). Based on these results, a CCE concentration range of 30–100 *μ*g/mL was chosen for subsequent experiments.

### 3.2. CCE Inhibits NO and PGE_2_ Production in LPS-Induced RAW264.7 Macrophages

To evaluate the effects of CCE on NO production in LPS-stimulated RAW264.7 cells, we measured nitrite levels using the Griess reagent. Cells were pretreated with CCE (30, 50, or 100 *μ*g/mL) for 1 h before adding LPS (100 ng/mL). Stimulation for 24 h with LPS resulted in a marked increase in NO levels. However, this increase was attenuated by CCE pretreatment in a concentration-dependent manner (Figure 1(b)). Additionally, we investigated the effect of CCE on the production of PGE_2_ using an ELISA in LPS-stimulated RAW264.7 cells. Cells were pretreated with CCE for 1 h and then stimulated with LPS (100 ng/mL) for 24 h. Pretreatment with the indicated concentrations of CCE resulted in a significant reduction in PGE_2_ production (Figure 1(c)).

### 3.3. CCE Attenuates the Expression of iNOS and COX-2 in LPS-Induced RAW264.7 Macrophages

**Figure 2:**
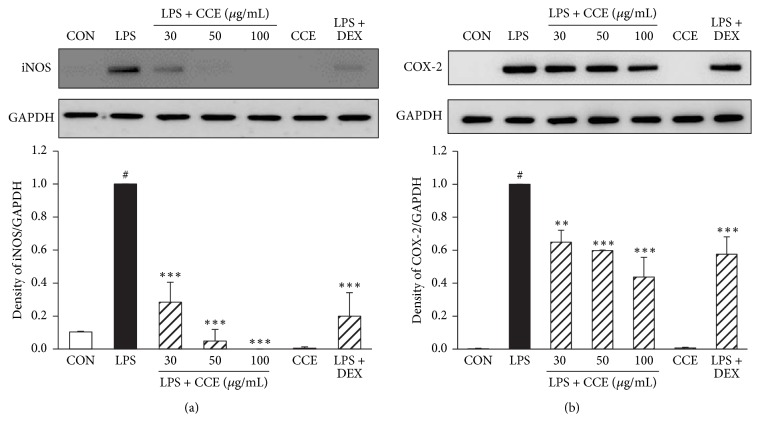
Effect of CCE on LPS-induced iNOS and COX-2 expression in RAW264.7 macrophages. RAW264.7 cells were pretreated with CCE (30–100 *μ*g/mL) for 1 h and stimulated with LPS (100 ng/mL). iNOS ((a), for 24 h) and COX-2 ((b), for 7 h) protein levels were analyzed in whole cell lysates using western blotting. GAPDH expression was used as an internal control. Each value indicates the mean ± SEM and is representative of results obtained from three independent experiments. ^#^*P* < 0.05 versus control group; ^*∗∗*^*P* < 0.01, ^*∗∗∗*^*P* < 0.001 versus LPS-stimulated group. DEX: dexamethasone.

The levels of NO and PGE_2_ are related to the activities of iNOS and COX-2, respectively; therefore, we evaluated the effects of CCE on the production of iNOS or COX-2 in LPS-induced RAW264.7 cells. Cells were treated with CCE (30, 50, or 100 *μ*g/mL) for 1 h before adding LPS (100 ng/mL). LPS stimulation for 24 h and 7 h markedly increased the expression of iNOS and COX-2, respectively. However, this high expression was significantly inhibited in cells pretreated with CCE in a concentration-dependent manner (Figure 2).

### 3.4. CCE Attenuates TNF-*α*, IL-6, and IL-1*β* Production in LPS-Induced RAW264.7 Macrophages

**Figure 3:**
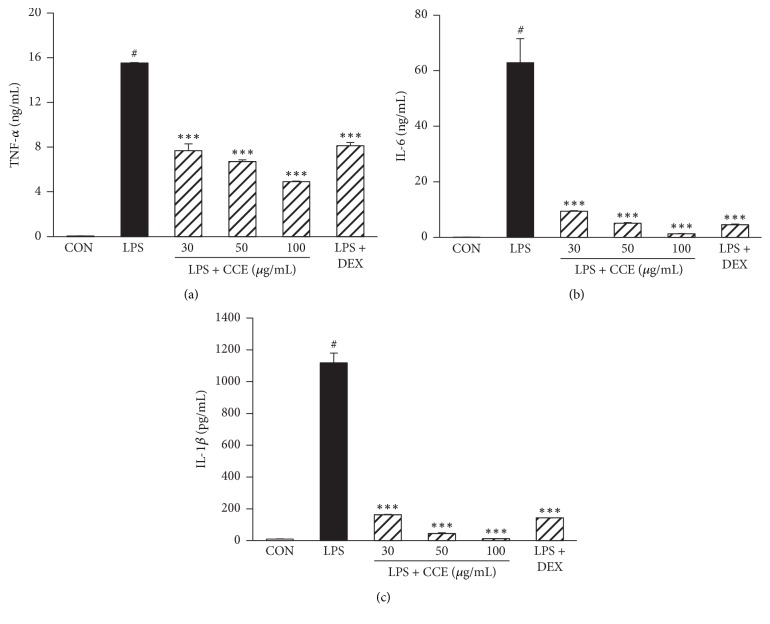
Effect of CCE on proinflammatory cytokine production in LPS-induced RAW264.7 macrophages. RAW264.7 cells were pretreated with CCE (30–100 *μ*g/mL) for 1 h and stimulated with LPS (100 ng/mL) for 24 h. Levels of TNF-*α* (a), IL-6 (b), and IL-1*β* (c) that were present in the supernatants were quantified using ELISA. Each value indicates the mean ± SEM and is representative of results obtained from three independent experiments. ^#^*P* < 0.05 versus control group; ^*∗∗∗*^*P* < 0.001 versus LPS-stimulated group. DEX: dexamethasone.

We investigated the effects of CCE on the expression of the proinflammatory cytokines TNF-*α*, IL-6, and IL-1*β* in LPS-induced RAW264.7 cells. RAW264.7 cells were incubated with the indicated concentrations of CCE (30, 50, or 100 *μ*g/mL) for 1 h in the presence or absence of LPS (100 ng/mL) for 24 h and TNF-*α*, IL-6, and IL-1*β* levels were measured in the culture medium. Stimulation of cells with LPS increased the levels of these cytokine. However, pretreatment with CCE significantly attenuated their expression in a concentration-dependent manner (Figure 3).

### 3.5. CCE Inhibits LPS-Stimulated Phosphorylation of MAPKs and Akt in RAW264.7 Macrophages

**Figure 4:**
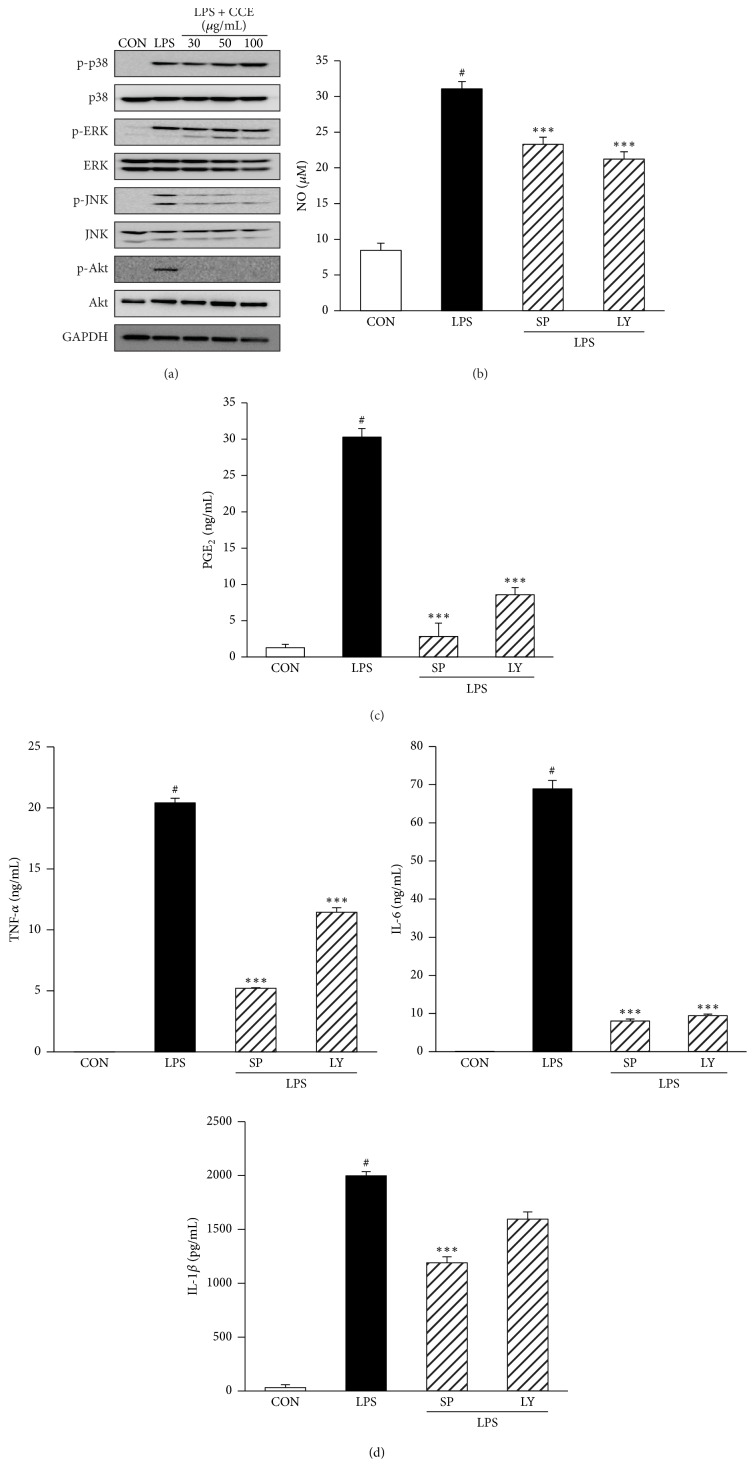
Effects of CCE on LPS-induced phosphorylation of MAPKs and Akt in RAW264.7 macrophages. RAW264.7 cells were pretreated with either the vehicle or the indicated concentration of CCE (30–100 *μ*g/mL) for 30 min, prior to stimulation with LPS (100 ng/mL). (a) Cell extracts were then prepared and subjected to western blotting with antibodies specific for the total and phosphorylated forms of MAPKs (p38, ERK1/2, JNK) (for 15 min) and Akt (for 4 h). The results are representative of three independent experiments. Levels of NO and PGE_2_ in the supernatant were detected using the Griess reaction assay (b) and ELISA (c). (d) The expression levels of TNF-*α*, IL-6, and IL-1*β* in the culture medium were determined using ELISA. Cells were treated with SP600125 (a JNK inhibitor) and LY294002 (an Akt inhibitor) 30 min prior to LPS stimulation for 24 h. Each value indicates the mean ± SEM and is representative of the results obtained from three independent experiments. ^#^*P* < 0.05 versus control group; ^*∗∗∗*^*P* < 0.001 versus LPS-stimulated group. SP: SP600125 and LY: LY294002.

Experiments were designed to investigate the signaling cascades that induce the expression of inflammatory mediators in RAW264.7 cells in response to stimulation by LPS. To investigate the mechanisms underlying the effects of CCE on the expression of inflammatory mediators, we examined the effect of CCE on the LPS-induced phosphorylation of MAPKs and Akt in RAW264.7 cells using western blotting. RAW264.7 cells were incubated with the indicated concentrations of CCE (30, 50, or 100 *μ*g/mL) for 1 h in the presence or absence of LPS (100 ng/mL). These proteins are phosphorylated following stimulation with LPS. Thus, we examined the effects of CCE on the LPS-induced activation of MAPKs and Akt. CCE markedly inhibited JNK and Akt activation but did not inhibit the activation of p38 and ERK (Figure 4(a)). Next, to verify whether the JNK and Akt signaling pathways are involved in the expression of inflammatory mediators, RAW264.7 cells were treated with LPS with or without JNK and Akt inhibitors. The upregulated expression of inflammatory mediators (NO, PGE_2_, IL-6, TNF-*α*, and IL-1*β*) was significantly inhibited by SP600125 (an inhibitor of JNK) and LY294002 (an inhibitor of Akt) (Figures 4(b), 4(c), and 4(d)).

### 3.6. CCE Inhibits LPS-Stimulated NF-*κ*B and AP-1 Activation in RAW264.7 Macrophages

**Figure 5:**
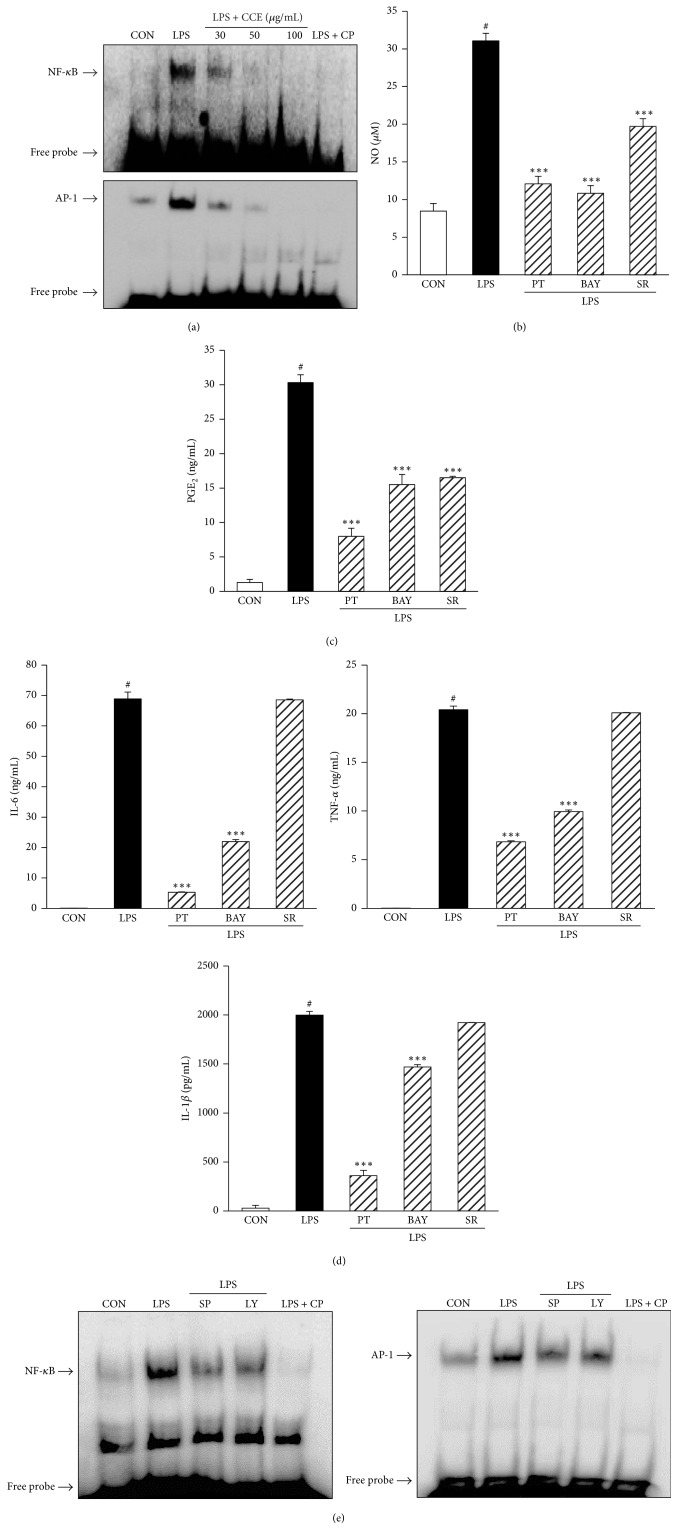
Effect of CCE on NF-*κ*B and AP-1 activation in LPS-induced RAW264.7 macrophages. (a) RAW264.7 cells were pretreated with CCE (30, 50, or 100 *μ*g/mL) for 1 h and then stimulated with LPS for 2 h. Nuclear extracts were prepared and evaluated for NF-*κ*B and AP-1 using EMSA. (b, c, and d) Cells were pretreated with NF-*κ*B inhibitors (BAY 11-7082 and parthenolide) and AP-1 inhibitor (SR-11032) for 30 min and were then stimulated with LPS (100 ng/mL) for 24 h. Levels of NO and PGE_2_ in the supernatant were detected using the Griess reaction assay (b) and ELISA (c). (d) The expression levels of TNF-*α*, IL-6, and IL-1*β* in the culture medium were determined using ELISA. Each value indicates the mean ± SEM and is representative of results obtained from three independent experiments. ^#^*P* < 0.05 versus control group; ^*∗∗∗*^*P* < 0.001 versus LPS-stimulated group. (e) Cells were pretreated with SP600125 (a JNK inhibitor) and LY294002 (an Akt inhibitor) 30 min prior to LPS stimulation for 2 h. Nuclear extracts were prepared and evaluated for NF-*κ*B and AP-1 using EMSA. All experiments were repeated three times and representative results are shown. CP: cold (unlabeled) probe, PT: parthenolide, BAY: BAY 11-7082, SR: SR-11032, SP: SP600125, and LY: LY294002.

The production of inflammatory mediators is regulated by nuclear factor-kappa B (NF-*κ*B) and activator protein-1 (AP-1) transcription factors in the inflammatory process [13–15]. Therefore, to assess the mechanism by which CCE affects the expression of inflammatory mediators, we examined the effects of CCE on the DNA-binding activity of NF-*κ*B and AP-1 using EMSA. We found that LPS stimulation significantly increased the DNA-binding activity of AP-1 and NF-*κ*B, whereas pretreatment with CCE significantly suppressed the LPS-induced DNA-binding activity of NF-*κ*B and AP-1 (Figure 5(a)). Next, to confirm whether the transcription factor signaling pathways for AP-1 and NF-*κ*B are involved in the expression of inflammatory mediators, RAW264.7 cells were treated with LPS with or without NF-*κ*B inhibitors (BAY 11-7082 and parthenolide) and an AP-1 inhibitor (SR 11032). Based on the results shown in Figures 5(b) and 5(c), we found that NF-*κ*B inhibitors and the AP-1 inhibitor suppressed the LPS-stimulated expression of NO and PGE_2_. However, the upregulated production of proinflammatory cytokines (TNF-*α*, IL-6, and IL-1*β*) by LPS stimulation was not inhibited by the AP-1 inhibitor (Figure 5(d)). To elucidate what upstream pathways control AP-1 and NF-*κ*B activation, we treated the cells with JNK and Akt inhibitors and used EMSA to assess AP-1 and NF-*κ*B activation. As shown in Figure 5(e), treatment with SP600125 or LY294002 markedly reduced the LPS-induced DNA-binding activities of AP-1 and NF-*κ*B.

## 4. Discussion

Marine algae represent a resource that may provide a large number of therapeutic agents with the potential to attenuate the production of inflammatory mediators in various inflammatory diseases [16]. Therefore, the present study was undertaken to evaluate the pharmacological activity of CCE on the production of proinflammatory mediators such as NO, PGE_2_, IL-1*β*, IL-6, and TNF-*α* by investigating inhibitory effects in LPS-stimulated murine macrophage RAW264.7 cells. To further understand the molecular mechanisms of CCE activity, we examined the effects of CCE on the phosphorylation of MAPKs and Akt and the activation of the transcription factor NF-*κ*B and AP-1. The results of this study show that CCE effectively attenuates LPS-induced production of inflammatory mediators through blockade of the JNK, Akt, NF-*κ*B, and AP-1 pathways in RAW264.7 macrophages. The inhibitory activity of CCE suggests one of the regulatory mechanisms responsible for its anti-inflammatory action and its potential utility to treat LPS-stimulated inflammatory diseases.

The overactivation of macrophages by inflammatory stimulants leads to the overproduction of proinflammatory mediators, including PGE_2_ and NO, and inflammatory cytokines, resulting in various chronic inflammatory diseases [17]. Thus, the elimination of the excessive expression of proinflammatory mediators is an effective therapeutic strategy to control chronic inflammation. In this study, we observed that CCE significantly attenuated the LPS-induced increase in proinflammatory mediators (NO, PGE_2_, TNF-*α*, IL-6, and IL-1*β*) without cytotoxicity in RAW264.7 macrophages (Figures 2 and 3). Thus, the results suggest that CCE is a promising agent for inhibiting inflammatory disorders.

The MAPKs (ERK-1/2, p38, and JNK) are members of proinflammatory signal transduction pathways that appear to play important roles in the inflammatory processes of LPS-activated macrophages [17]. Phosphatidylinositol 3-kinase/Akt (PI3K/Akt) also contributes to the induction of proinflammatory factors [18]. Therefore, application of anti-inflammatory agents that effectively modulate MAPK and Akt pathways are good approaches to treat various inflammatory disorders. To understand the signaling mechanisms associated with CCE-mediated regulation of proinflammatory mediator expression, the effect of CCE on the phosphorylation of MAPKs and Akt was evaluated (Figure 4). As expected, when macrophages were stimulated with LPS, MAPKs and Akt were phosphorylated; however, pretreatment with CCE significantly attenuated LPS-induced phosphorylation of JNK and Akt, but not p38 and ERK. CCE showed a preference for p-JNK, without affecting p-ERK or p-p38 in the MAPK signaling pathway. In other words, CCE targeted only p-JNK. To confirm the above results, we investigated the effects of JNK and Akt inhibitors (SP600125 and LY294002, resp.) on the LPS-induced production of proinflammatory mediators by macrophages. Results showed that CCE inhibits the production of NO, PGE_2_, TNF-*α*, IL-6, and IL-1*β* in LPS-stimulated macrophages by suppressing the activation of JNK and Akt, consequently attenuating the expression of proinflammatory mediators. These results suggested that CCE could inhibit LPS-induced inflammatory responses that are mediated by the JNK and Akt signaling pathways.

Previous studies reported that the transcription factors NF-*κ*B and AP-1 regulate the production of many inflammatory mediators [13, 14]. Therefore, in this study, we evaluated the effects of CCE on the LPS-stimulated activation of NF-*κ*B and AP-1 in macrophages. Activated transcription factors enter the nucleus and induce inflammatory mediator production; therefore, we examined the DNA-binding activity of NF-*κ*B and AP-1. Studies using EMSA revealed that stimulation with LPS increased the DNA-binding activities of NF-*κ*B and AP-1 in the nuclear compartment. However, pretreatment with CCE attenuated the induced DNA-binding activities of the transcription factors in a concentration-dependent manner (Figure 5(a)). To confirm the anti-inflammatory activity of CCE via regulation of the binding activities of transcription factors, we examined the effects of BAY 11-7082, parthenolide (NF-*κ*B inhibitors) and SR11302 (an AP-1 inhibitor) on the production of inflammatory mediators in LPS-stimulated macrophages. As shown in Figures 5(b)–5(d), we found that the NF-*κ*B inhibitors attenuated LPS-induced production of NO, PGE_2_, IL-6, TNF-*α*, and IL-1*β* in the macrophages. Additionally, the AP-1 inhibitor blocked LPS-induced NO and PGE_2_ production. However, interestingly, we found that proinflammatory cytokines (IL-6, TNF-*α*, and IL-1*β*) were not inhibited by treatment with the AP-1 inhibitor (Figure 5(e)). This result showed that the production of proinflammatory cytokines, such as IL-6, TNF-*α*, and IL-1*β*, is not directly related to the AP-1 signaling pathway. Previous reports showed that the expression of inflammatory cytokines is regulated by activation of the AP-1 transcription factor [19–21]. However, since these previous studies did not confirm this finding using an AP-1 inhibitor, the results that we obtained would not have been observed in the previous studies. To the best of our knowledge, our AP-1 inhibitor results demonstrated, for the first time, that LPS directly stimulates the production of inflammatory mediators in RAW264.7 macrophages. In addition, using inhibitors of JNK and Akt, we showed that LPS stimulation attenuates the activation of NF-*κ*B and AP-1. It has been shown that the JNK and Akt signaling pathways can be upstream activators of the NF-*κ*B and AP-1 signaling cascade. Based on the above results, we demonstrated that LPS induced NF-*κ*B and AP-1 activation in RAW264.7 macrophages via upstream signaling molecules that are involved in the JNK and Akt pathways.

The present study demonstrated that CCE exerts inhibitory effects on NO and PGE_2_ production in the LPS-induced RAW264.7 macrophages. Furthermore, CCE markedly attenuated the expression of proinflammatory cytokines, including IL-1*β*, IL-6, and TNF-*α*. These effects of CCE are associated with the inactivation of LPS-induced NF-*κ*B and AP-1 via inhibition of JNK and Akt phosphorylation. In particular, we have found, for the first time, that the production of proinflammatory cytokines, such as IL-6, TNF-*α*, and IL-1*β*, is not directly related to the AP-1 signaling pathway. Thus, we concluded that CCE possesses potential anti-inflammatory activities and may have therapeutic benefits in the treatment of various inflammatory disorders. Further studies are needed to investigate the components of CCE that may have beneficial and physiologically active effects.
